# Intravesical Mesh Migration After Hernia Repair: A Case Report

**DOI:** 10.15190/d.2026.9

**Published:** 2026-05-31

**Authors:** Syed Muhammad Hadi Ali Shah, Maheen Nasir, Fahad Malik, Shumaila Seemi Malik, Safdar Ali Malik

**Affiliations:** ^1^Al Noor Diagnostic Center, Pakistan; ^2^CMH Lahore Medical and Dental College, Pakistan

**Keywords:** Inguinal hernia, intravesical mesh, hernia repair.

## Abstract

While the recurrence rates of inguinal hernia have been significantly reduced by the use of mesh in laparoscopic hernia repair, it has also introduced rare complications, including the uncommon erosion of the mesh into adjacent viscera. We report a 36-year-old male, who following left-sided hernia repair in 2014, presented with persistent dysuria, increased urinary frequency and chronic discomfort at the surgical site. Initially, his symptoms were intermittent but gradually progressed to recurrent urinary tract infections unresponsive to prolonged antibiotic therapy. On further investigation, ultrasound revealed an echogenic linear structure extending from the left inguinal region into the urinary bladder, and contrast-enhanced CT confirmed mesh migration, along with a pseudo-diverticular outpouching from the bladder wall. The imaging was corroborated by surgical findings, and the mesh was removed. This case adds to the limited existing literature and emphasizes the importance of considering mesh-related complications in patients with persistent urinary symptoms after hernia repair. Additionally, it highlights the key role of imaging in diagnosis and in guiding management.

## INTRODUCTION

Evolving surgical techniques have clear advantages, including reduced recurrence rates and decreased post-operative complications; however, they are not without unique complications. The posterior approach to inguinal hernia (IH) repair, particularly via laparoscopy, is associated with a distinct spectrum of adverse outcomes, including but not limited to mesh-related complications^1^. Among these, mesh erosion into adjacent viscera represents a rare and unique entity. While erosion of the mesh into adjacent viscera, such as the bowel, has been more frequently described in the literature, involvement of the urinary bladder (UB) remains an uncommon and underreported phenomenon^2^. A comprehensive review of the literature spanning three decades yields only a handful of documented cases of erosion of the mesh into the urinary bladder. Because the condition is so seldom encountered, it is rarely considered in the differential diagnosis when patients present with lower urinary tract symptoms following herniorrhaphy. This diagnostic blind spot can lead to prolonged morbidity before the true source is identified. 

 We present the case of a young male who, following laparoscopic totally extraperitoneal (TEP) repair for inguinal hernia, developed a delayed erosion of mesh into the urinary bladder, which manifested as persistent urinary symptoms and recurrent infections. The diagnosis was then established on imaging and confirmed intraoperatively. This case aims to bring to light the need for a high index of suspicion for rare mesh-related complications and additionally, emphasizes the complementary role of imaging and surgical evaluation in achieving an accurate diagnosis and appropriate management. With so few precedents in the literature, the present case adds meaningfully to the limited body of evidence on this complication.

## CASE PRESENTATION

A 36-year-old male underwent laparoscopic totally extraperitoneal (TEP) repair for inguinal hernia at a private hospital in 2014. Shortly after the procedure, he started to experience discomfort accompanied by a pinching pain at the surgical site. He was reassured, and his discomfort was managed conservatively using analgesics. Approximately 6 to 12 months after surgery (2015), he developed his first episode of urinary tract infection, characterized by painful, burning micturition and increased urinary frequency. He subsequently experienced recurrent episodes of UTIs over the following years and received multiple courses of antibiotics along with local remedies. He noticed partial improvement in his symptoms with treatment; however, his symptoms recurred intermittently over time. 

 In December 2021, nearly seven years after the initial hernia repair, he presented to the emergency department with a severe urinary tract infection. Urine culture revealed growth of Escherichia coli and Pseudomonas, and he was treated with culture-directed antibiotics. Despite prolonged antibiotic therapy for around eight to twelve weeks, his symptoms persisted. He was later referred for imaging and thus underwent an ultrasound examination. 

**Figure 1:**
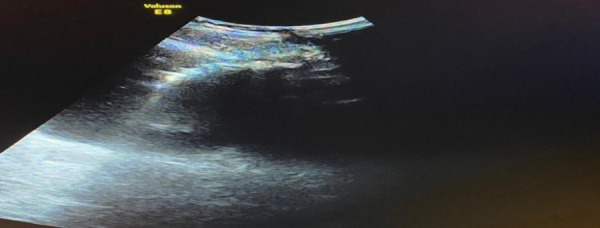
Ultrasound Pelvis showing hernia mesh projecting in urinary bladder

**Figure 2:**
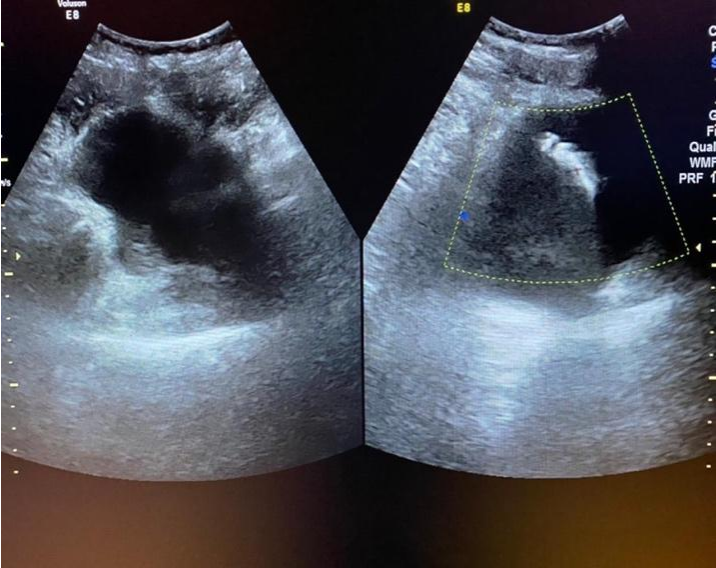
Ultrasound of bladder showing hernial mesh projecting into urinary bladder On Color Doppler, no significant blood flow seen

 The ultrasound revealed an echogenic linear structure within the anterior abdominal wall in the left inguinal region, with extension into the urinary bladder through the left anterolateral wall of the bladder. Approximately, half of the mesh was seen to be located within the abdominal wall, while the remainder was visualized within the lumen of the bladder, as shown in **Figure 1** and **Figure 2**. Additionally, no blood flow was seen on the Color Doppler.

**Figure 3:**
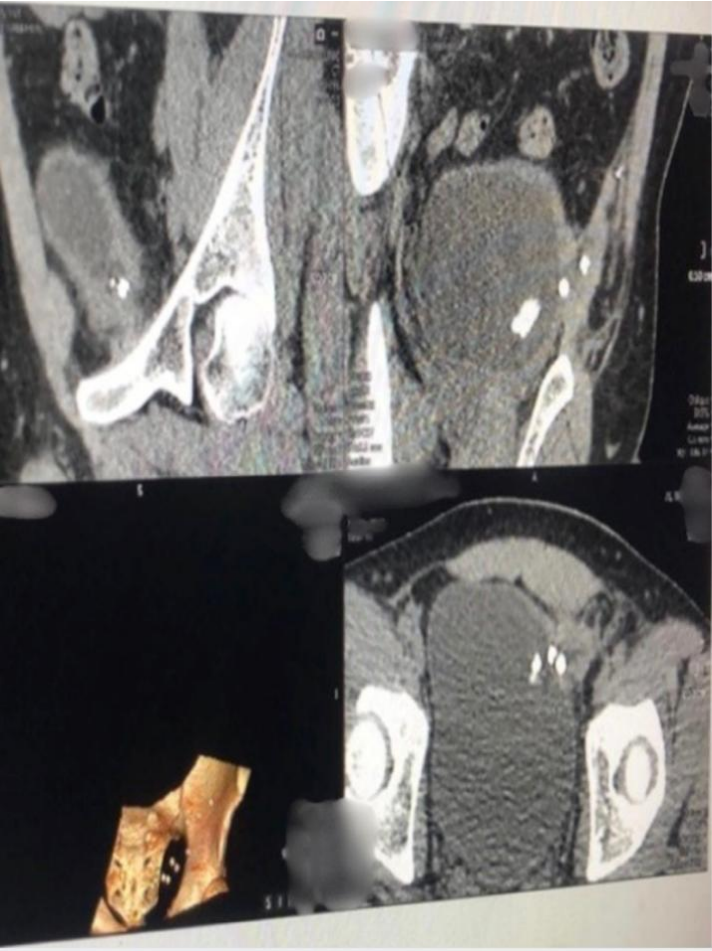
Contract enhanced CT Pelvis sagittal, coronal, axial and 3D reformats showing hernial mesh projecting into urinary bladder

**Figure 4:**
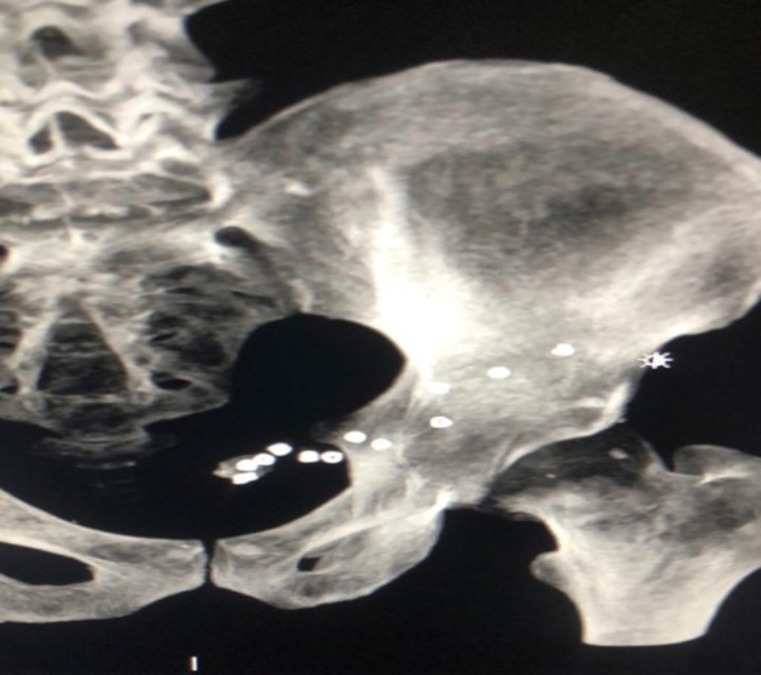
Zoomed view of 3D pelvis showing hernial mesh in pelvis

The findings of the ultrasound were further evaluated using contrast-enhanced CT (CECT) scan of the abdomen and pelvis (**Figure 3** and **Figure 4**). The CECT revealed a pseudo-diverticular outpouching arising from the left anterolateral wall of the urinary bladder. The outpouching was visualized to be extending laterally beneath the rectus muscle toward the anterior pelvic wall, closely abutting the left inguinal canal. On the CECT, the outpouching appeared to be surrounded by dense fibrous tissue and contained multiple hyperdense foci, consistent with displaced mesh material within both the outpouching and the bladder cavity.

**Figure 5:**
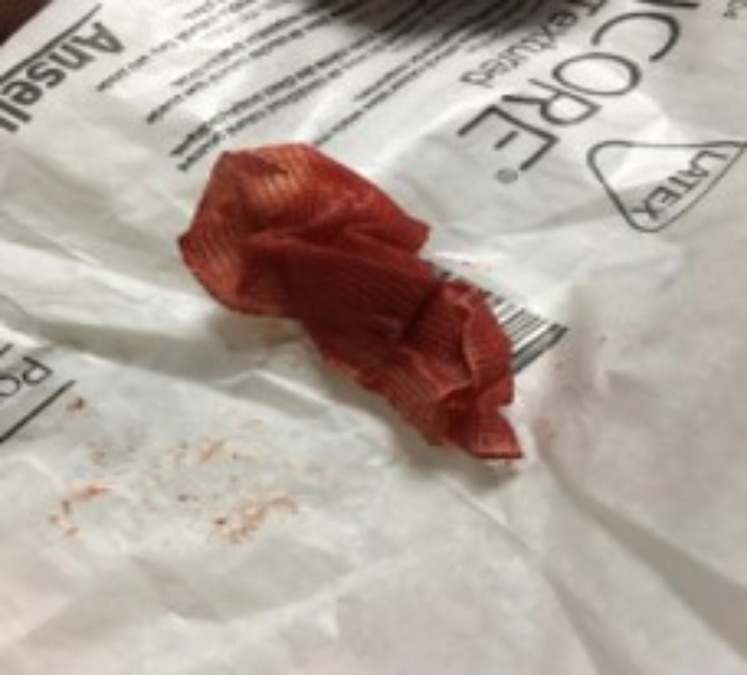
Hernial mesh removed during surgery

Cystoscopy was not performed preoperatively. In this case, the combined ultrasound and contrast-enhanced CT findings demonstrated clear evidence of mesh migration with intravesical extension and associated inflammatory changes, which were considered sufficient to procedd with surgical exploration and definitive management. The diagnosis was subsequently confirmed intraoperatively, and the removed mesh is shown in **Figure 5**. 

Surgical exploration revealed the erosion of the mesh into the urinary bladder, necessitating complete excision of the mesh followed by primary repair of the bladder defect in two layers using 2.0 Vicryl absorbable sutures. Postoperatively urinary drainage was maintained with a Foley catheter for adequate bladder healing. The patient had an uneventful recovery and a post-operative course. He experienced complete resolution of his symptoms and follow-up CT imaging demonstrated resolution of the previously noted outpouching, with no residual foreign material or evidence of further complications.

## DISCUSSION

Mesh-related complications following inguinal hernia repair are rare and most commonly occur with placement of the mesh within the preperitoneal space. Among these, migration and subsequent erosion of the mesh into adjacent viscera are uncommon but clinically significant complications, as they may further lead to the formation of a fistula. It is important to note that such complications are rarely reported following anterior repairs, and when described, are found to be because of the use of mesh plug with eventual migration^1^. 

 Multiple factors contribute to the migration of the mesh, with surgical technique playing a central role. Importantly, inadequate fixation, improper placement, or failure of mesh anchoring are among the key issues that may cause eventual complications. Additionally, a review of the literature revealed that mesh migration is more commonly observed following the transabdominal preperitoneal (TAPP) approach^3,4^. Additional contributing mechanisms include other intraoperative technical errors such as improper suturing and foreign body reactions, which lead to gradual erosion of the tissue and can cause displacement of the mesh^5^.

 Erosion of the mesh into the urinary bladder may present with a range of non-specific clinical symptoms. Erosion may cause recurrent urinary tract infections, and patients may present with dysuria, hematuria, or increased urinary frequency. In some cases, the presentation may be even more subtle or clinically vague. This includes presenting with features mimicking a vesical calculus or with complications of the erosion, such as a vesicocutaneous fistula^6^. These non-specific features highlight the need for a high index of clinical suspicion, particularly in patients with a history of laparoscopic inguinal hernia repair who present with persistent and unexplained urinary symptoms. This is particularly relevant in younger patients, as shown in our case. Similar findings were also reported by Agrawal and Avill, where cystoscopic evaluation ultimately established the diagnosis^7^.

 Initial evaluation includes urine culture and microscopy to confirm infection and identify the causative organism. Ultrasound is often the first-line imaging modality and may demonstrate an echogenic linear or intravesical structure, raising suspicion of a foreign body within or adjacent to the urinary bladder. A focal opacity in the bladder region, in case of calcification of the eroded mesh, may be visualized on plain radiography of the kidney, ureter, and bladder (KUB). Advanced imaging, especially contrast-enhanced CT urography, play a pivotal role as it may help in delineating the thickening of the bladder wall and the presence of any foreign material within the bladder^8^. Additionally, it may help identify any fistulous tracts. However, the definitive modality remains cystoscopy as it allows the direct visualization of the eroded mesh, which is often partially obscured by encrustations^9^. 

 Definitive management involves surgical interventions for complete removal of the mesh, typically performed via an extraperitoneal approach. In addition to mesh removal, the bladder defect should be carefully identified and debrided to healthy margins. It should then be repaired in a layered fashion. Urinary diversion is often required which is accomplished using suprapubic or urethral catheterization. Additionally, adequate drainage of the space of Retzius promotes healing and prevents complications, ensuring an uneventful post-operative course^10^. 

## CONCLUSION

Mesh erosion into adjacent viscera, particularly the bladder, is an increasingly recognized but rare complication of laparoscopic inguinal hernia repair. Therefore, a high degree of clinical suspicion is essential to identify the mesh-related complications. Early diagnosis through proper imaging and timely surgical intervention can significantly reduce morbidity and lead to a resolution of symptoms. This case further contributes to the growing body of literature on this uncommon complication and reinforces the importance of multidisciplinary evaluation in achieving optimal outcomes.

## Acknowledgements

Written informed consent was obtained from the patient for publication of this case report and any accompanying images. All procedures performed were in accordance with the ethical standards of the institution. There was no external funding for this work.

## Declaration of Generative AI and AI-assisted technologies

This work was done without the use of artificial intelligence (AI). No element of manuscript development involved AI.

## Publisher’s note 

All claims expressed in this article are solely those of the authors and do not necessarily represent those of their affiliated organizations, or those of the publisher, the editors and the reviewers. Any product that may be evaluated in this article, or claim that may be made by its manufacturer, is not guaranteed or endorsed by the publisher.
